# An Online Tool Using Basal or Activated Ovarian Reserve Markers to Predict the Number of Oocytes Retrieved Following Controlled Ovarian Stimulation: A Prospective Observational Cohort Study

**DOI:** 10.3389/fendo.2022.881983

**Published:** 2022-05-27

**Authors:** Yong Han, Huiyu Xu, Guoshuang Feng, Kannan Alpadi, Lixue Chen, Haiyan Wang, Rong Li

**Affiliations:** ^1^ Department of Thoracic Surgery, Zhejiang Provincial People’s Hospital, Affiliated People’s Hospital, Hangzhou Medical College, Hangzhou, China; ^2^ Key Laboratory of Tumor Molecular Diagnosis and Individualized Medicine of Zhejiang Province, Hangzhou, China; ^3^ Center for Reproductive Medicine, Department of Obstetrics and Gynecology, Peking University Third Hospital, Beijing, China; ^4^ Key Laboratory of Assisted Reproduction (Peking University), Ministry of Education, Beijing, China; ^5^ National Clinical Research Center for Obstetrics and Gynecology (Peking University Third Hospital), Beijing, China; ^6^ Beijing Key Laboratory of Reproductive Endocrinology and Assisted Reproductive Technology, Beijing, China; ^7^ Big Data Center, Beijing Children’s Hospital, Capital Medical University, National Center for Children’s Health, Beijing, China; ^8^ The Predict Health, Houston, TX, United States

**Keywords:** predicting model, NORs, negative binomial regression, pruned forward selection with holdback validation, online tool

## Abstract

**Background:**

Predicting the number of oocytes retrieved (NOR) following controlled ovarian stimulation (COS) is the only way to ensure effective and safe treatment in assisted reproductive technology (ART). To date, there have been limited studies about predicting specific NOR, which hinders the development of individualized treatment in ART.

**Objective:**

To establish an online tool for predicting NOR.

**Materials and Methods:**

In total, 621 prospective routine gonadotropin releasing hormone (GnRH) antagonist COS cycles were studied. Independent variables included age, body mass index, antral follicle counts, basal FSH, basal and increment of anti-mullerian hormone, Luteinizing hormon, estradiol, testosterone, androstenedione, and inhibin B. The outcome variable was NOR. The independent variables underwent appropriate transformation to achieve a better fit for a linear relationship with NOR. Pruned forward selection with holdback validation was then used to establish predictive models. Corrected Akaike’s information criterion, Schwarz–Bayesian information criterion, scaled –*log*[likelihood], and the generalized coefficient of determination (*R* ^2^) were used for model evaluation.

**Results:**

A multiple negative binomial regression model was used for predicting NOR because it fitted a negative binomial distribution. We established Model 1, using basal ovarian reserve markers, and Model 2, using both basal and early dynamic markers for predicting NOR following COS. The generalized *R* ^2^ values were 0.54 and 0.51 for Model 1 and 0.64 and 0.62 for Model 2 in the training and validation sets, respectively.

**Conclusion:**

Models 1 and 2 could be applied to different scenarios. For directing the starting dose of recombinant follicle stimulation hormone (rFSH), Model 1 using basic predictors could be used prior to COS. Model 2 could be used for directing the adjustment of rFSH dosages during COS. An online tool (http://121.43.113.123:8002/) based on these two models is also developed. We anticipate that the clinical application of this tool could help the ART clinics to reduce iatrogenic ovarian under- or over-responses, and could reduce costs during COS for ART.

## Introduction

The number of oocytes retrieved (NOR) is acknowledged to be a robust surrogate prognostic marker for successful pregnancy in women undergoing controlled ovarian stimulation (COS) following *in-vitro* fertilization (IVF) or intracytoplasmic sperm injection (ICSI) cycles in assisted reproductive technology (ART). Optimal NOR contribute to an increase in the live birthrate (LBR) in ART clinics (1–4). In an analysis of 400,135 fresh IVF/ICSI cycles, the LBR increased up to ∼15 oocytes recovered, plateaued at 15–20, and declined steadily at >20. The results showed a nonlinear relationship between NOR and LBR, with a maximum LBR at ∼15 retrieved oocytes. It is not desirable to have either too few NOR, known as an poor ovarian response (under-response) to COS, or too many (over-response) (1).

Predicting the NOR before COS is the only way to ensure effective and safe ART outcomes. Various markers have been used to assess the ovarian response, including the woman’s age, the antral follicular count (AFC), and basal follicle-stimulating hormone (FSH), inhibin B, and anti-Müllerian hormone (AMH) levels (5–8), of which the latter is considered as the most efficient marker for predicting ovarian response (5). Thus far, no single ovarian reserve marker has shown superior accuracy in this regard (9). Clinicians often choose the starting dose of recombinant follicle stimulating hormone (rFSH) according to their clinical experience based on each woman’s history of ovarian response, age, AMH level, the AFC, basal FSH level and body mass index (BMI) among other factors. However, until now the selection of the starting dose of rFSH and adjusting the rFSH dosage during COS mainly relies on each clinician’s experience, and there are no acknowledged criteria or protocols.

In this study, we first planned to use basic ovarian reserve markers measured prior to COS to establish a model for predicting NOR. Ideally, such a model could be used to guide the starting dose of rFSH. However, even subjects with the same ovarian reserve have great differences in their responsiveness to COS. We next explored the combination of both basic and dynamic factors during COS to establish a new model for predicting NOR, anticipating that this new model could be used to adjust rFSH doses. We hope that the clinical application of our models could reduce iatrogenic ovarian under- or over-responses, and could also reduce the cost of COS during ART cycles.

## Materials and Methods

### Study Cohort

In this prospective study we analyzed the basal and dynamic changes of ovarian reserve markers to establish a model for predicting NOR. Data were collected from April to September 2020. Ethics approval was acquired from the institutional review board of Peking University Third Hospital, with granted number of 2015sz-017. Basic and clinical characteristics of all classical gonadotropin releasing hormone (GnRH)-antagonist cycles were recorded prospectively, without using any inclusion or exclusion criteria. The project was registered on the Chinese Clinical Trial Registry website (https://www.chictr.org.cn/abouten.aspx), with the registration number ChiCTR-OPC-16009002.

### Sampling and Endocrine Assays

Venous blood samples were drawn on days 2 and 6 of the menstrual cycle during COS. The tests on day 2 included assays for AMH, inhibin B, FSH, LH, estradiol (E_2_), progesterone (P), testosterone (T) and androstenedione (A4), and the same tests were run on day 6 except for FSH.

Serum FSH, LH, E2, T, and A4 measurements were performed using a Siemens Immulite 2000 immunoassay system (Siemens Healthcare Diagnostics, Shanghai, P. R. China). The quality controls for these assays were supplied by Bio-Rad (Lyphochek Immunoassay Plus Control, Trilevel, catalog number 370, lot number 40370; Bio-Rad Laboratories Inc., Hercules, CA, USA). Serum AMH and inhibin B concentrations were measured using an ultrasensitive two-site enzyme-linked immunosorbent assay (ELISA; Ansh Laboratories, Webster, TX, USA), using quality controls supplied with the kits. Trilevel or two-level controls of coefficients of variation of assays were <5% for AMH, inhibin B, FSH and LH, and <10% for E2, T, and A4, respectively.

### COS Treatment

Exogenous FSH used in our study include recombinant human FSH for injection (Gonal-F alfa from Merck Serono, Darmstadt, Germany; Puregon beta from MSD Kenilworth, NJ, USA), highly purified Urofollitropin for injections (Livzon Pharmaceutical Group Inc., P. R. China) and highly purified urinary human menopausal gonadotropins (HMGs) for injections (Livzon Pharmaceutical Group Inc., P. R. China). A standard GnRH-antagonist COS protocol was performed as follows: exogenous FSH administration was initiated on day 2 of the menstrual cycle. The starting dose was selected based on the woman’s age, AMH, basal FSH, AFC, BMI, and the results of previous COS attempts. The adjustment for exogenous FSH dose was further performed according to the number and size of growing follicles and serum E_2_ level on day 6 of COS. The GnRH antagonist (Ganirelix, MSD Kenilworth, NJ, USA; Cetrorelix, Merck Serono, Darmstadt, Germany) treatment was initiated when the growing follicles reached 10–12 mm in diameter by ultrasonography.

When at least two dominant follicles reached more than 18 mm in diameter, human chorionic gonadotropin (hCG; Choriogonadotropin alfa, Merck Serono) of 5000–10000 IU was injected to trigger oocyte maturation. For those with a high risk of Ovarian Hyperstimulation Syndrome, the trigger protocol was a GnRH antagonist alone or combined with 2000 IU exogenous hCG. Oocyte retrieval was carried out 36–38 h after hCG administration. One to two embryos were either transferred to the mother in fresh embryo transfer cycles or were cryopreserved for future thaw cycle. Luteal phase progesterone support (Progesterone Vaginal Gel, Merck Serono) was administered to the women thereafter.

### Definition of Different Causes of Infertility in Our Study

Male factor infertility was defined according to the WHO manual for the standardized diagnosis of the infertile couple^18^. Endometriosis was defined by the presence of endometrial glands and stroma outside the uterine cavity, with a combination of dysmenorrhea and dyspareunia. Polycystic ovary syndrome was defined according to the Rotterdam criteria^19^. Tubal factor infertility was diagnosed by laparoscopic examination when fallopian tube infertility was indicated by ultrosonic examination. The couples whose standard examinations, such as tests of ovulation, tubal patency and semen analysis, were normal, with a repeated failed pregnancy after sexual intercourse or intra-uterine insemination were classified into unexplained infertility.

### Statistical Analysis

In this study, the outcome variable was the specific number of NOR. Predictive variables in Model 1 using basal predictors included age, BMI, AFC, day 2 levels of AMH, FSH, LH, E_2_, T, A4, and inhibin B. Model 2 included all the predictors in Model 1 as well as their early dynamic levels (day 6 minus day 2 levels).

Exploration of the distribution of the best fit of model prediction with actual NOR was performed to discover the appropriate regression method. Then, the predictive variables were transformed appropriately according to functional forms, to make the predictive variables and outcome variate conform to a linear correlation. We used model-checking techniques based on cumulative residuals to assess appropriate functional forms of covariates and link functions (10). The pruned forward selection with holdback validation method was then used to establish a predictive model. For this, a specified proportion (70%) of the data was selected randomly as a training set for model fitting, and the rest (30%) of the data was used as the validation set. The scaled negative loglikelihood (–*Log* L *(β)*) was used to evaluate the final model: the smaller the value of scaled –*Log* L (*β*) in the validation set the better the model fit. Finally, a comparison of the models was assessed by scaled –*Log* L (*β*), corrected Akaike’s information criterion (AICc), Schwarz Bayesian information criterion (BIC), and the generalized coefficient of determination (*R* ^2^). Models with smaller scaled –*Log* L (*β*), AICc, and BIC values, and larger generalized *R* ^2^ are deemed better. To describe the contribution of each variable to NOR, the main effect and total effect for each variable were estimated by independent Monte Carlo sampling. In this, the main effect reflects the relative contribution of the variable alone, and the total effect represents the relative contribution of that variable both alone and in combination with other variables. Data were analyzed with JMP PRO v. 16.0 software from SAS Institute Inc. (Cary. NC, USA), and a two-sided P value of <0.05 was considered statistically significant.

## Results

We collected 669 GnRH antagonist COS cycles prospectively without data selection. After excluding 48 cycles without AFC or NOR recorded, a total of 621 cycles were finally included for analysis. Basal hormone levels on day 2 and activated hormone levels on day 6 were included. The dynamic change (Δ) level means day 6 minus day 2 level. The basal characteristics dynamic hormone levels and the clinical outcomes of ovarian stimulations were shown in **Table 1**.

**Table 1:** Ovarian reserve markers of basal (day-2) and dynamic (day-6 minus day-2) levels in classical GnRH-antagonist cycles.

	ovarian reserve markers		Ovarian stimulation outcomes
	Day-2 levels	Δ levels	
Age (years)	33(30-36)	–	
BMI (kg/m^2^)	21.9 (20.0-24.5)	–	
FSH (IU/L)	6.26 (5.16-7.93)	–	
LH (IU/L)	3.43 (2.43-4.76)	–1.94 (–2.99 to –1.05)	
E2 (pmol/L)	151 (121-176)	1113 (474-2093)	
AMH (ng/mL)	3.02 (1.63-5.33)	–0.5 (–1.21 to –0.16)	
Inhibin B (pg/mL)	87.9 (62.7-114.0)	642 (309-1172)	
T (nmol/L)	0.69 (0.69-0.80)	0 (0-0.15)	
A4 (nmol/L)	6.58 (4.96-9.21)	0.87 (–0.74 to 2.87)	
Main cause of infertility			
Male factor	184	–	
Endometriosis	39	–	
PCOS	102	–	
Tubal factor	167	–	
Unexplained and others	129	–	
NOR	–	–	12 (7-17)
2PN fertilization rate	–	–	0.60 (0.43-0.77)
Number of embryos available for transfer per cycle	–	–	3 (2-6)
The number of cycles underwent fresh embryo transfer	–	–	256
day 3 embryo transfer	–	–	248
day 5 embryo transfer	–	–	8
The number of cycles not underwent fresh embryo transfer	–	–	365

Key: Value represented as median (lower - upper quartiles);D levels, dynamic levels of day-6 minus day-2 of different ovarian reserve markers; PCOS, polycystic ovary syndrome; NOR, the number of oocytes retrieved; BMI, body mass index; T, testosterone; A4, androstenedione; –, not applicable; PN, pronuclear.

### Exploring the Distribution of Outcome Variable

**Figure 1:**
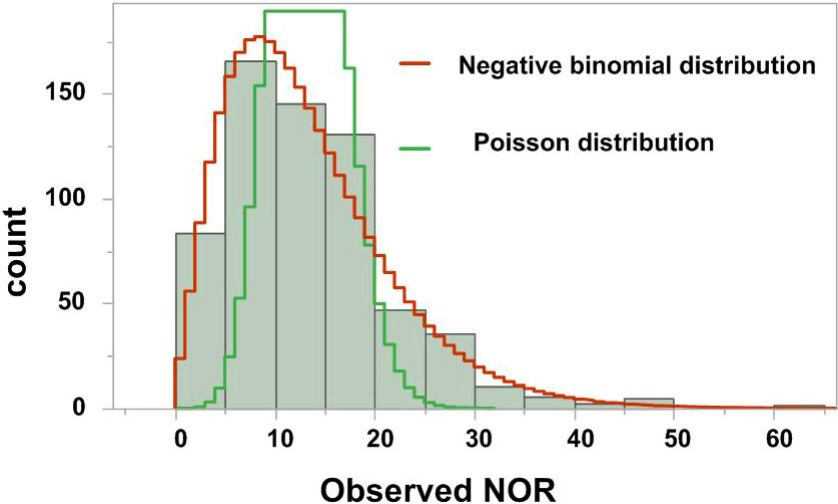
Distribution of the number of oocytes retrieved (NOR).

It is essential to explore the distribution of the outcome variable in such models. Once the distribution of the outcome variable is identified, one can then decide which statistical analysis is appropriate. The goodness-of-fit for Poisson distribution or negative binomial distribution for the outcome variable of NOR was tested. As shown in **Figure 1**, the distribution of the NOR was more similar to a negative binomial distribution than to a Poisson distribution, so the negative binomial regression was used for further analyses.

### Exploring the Functional Forms of Predicting Variables

**Figure 2:**
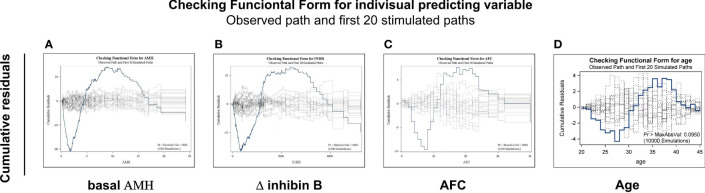
Exploring the functional forms of each predictive variable based on the cumulative sums of residuals. Functional forms of AMH **(A)**, Δinhibin B **(B)**, AFC **(C)** and age **(D)** based on the cumulative sums of residuals. The heavy line represents the observed cumulative residuals and the light line represents the stimulated theoretical cumulative residuals. If the heavy line deviated from the 10,000 light curves, a more complicated form instead of a linear one is needed for the predictive variables. Only first 20 simulated paths are shown in each figure.

The functional form of each possible predictive was explored for linear or other complicated forms. **Figure 2** presents the functional forms of selected predictive variables based on the cumulative sums of residuals. The heavy line represents the observed cumulative residuals, and the light line represents 10,000 stimulated theoretical cumulative residuals (only the first 20 paths are shown). If the heavy line deviates from the light curves, a more complicated form instead of a linear one is needed for the predictive variables. As shown in **Figure 2**, the variables basal AMH, Δ inhibin B and AFC deviated widely from the simulated theoretical curves. Then, logarithmic transformation of AMH, Δ inhibin B and AFC were carried out, with *R* ^2^ values of 0.30–0.52, 0.37–0.56, and 0.29–0.35, before and after transformation, respectively.

### Model 1 Using Basal Indicators Measured Before Ovarian Stimulation to Predict NOR

**Figure 3:**
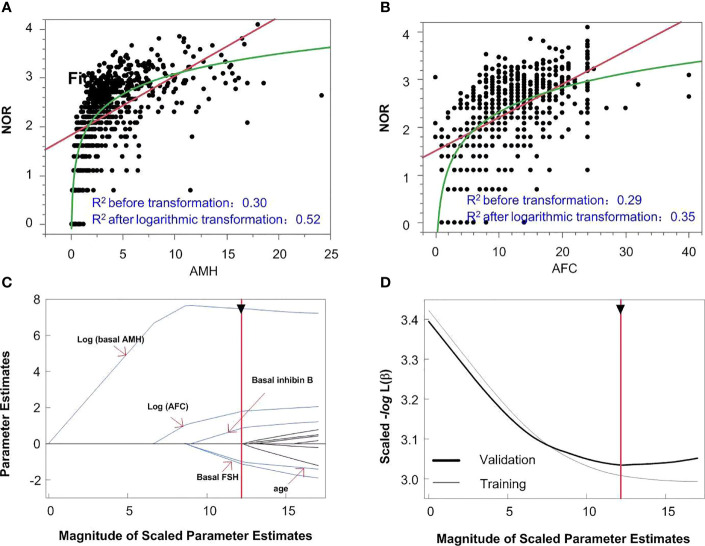
The model building process of Model 1. logarithmic or linear correlation between independent variables of AMH **(A)** and AFC **(B)** and outcome variables are shown in panels A and **(B)** The variable selection process is shown in panels **(C, D)** When five variables were included **(C)**, the scaled -*Log* L *(β)* in the validation set no longer decreased **(D)**.

The logarithmic relationship between independent variables (AMH and AFC) and the outcome variable were shown in **Figures 3A, B**, while the other independent variables were left in their original forms. The variable selection process is shown in **Figures 3C, D**. When five variables were included (**Figure 3C**), the scaled -*Log* L (*β*) value in the validation set no longer decreased (**Figure 3D**); thus, the five variables of log (basal AMH), log (AFC), basal FSH, age and basal inhibin B were finally included in Model 1. **Figure 3C** shows the order in which variables were included, starting with the most significant ones. The parameter estimation, main effect and total effect of each predictor in Model 1 are shown in **Table 2**. To be specific, if the value of a parameter estimation is positive, the NOR increase as well, and if it is negative, the NOR increase with a *decrease* in this predictive variable.

**Table 2:** Model-1 using basal predictors to predict the NOR by multiple negative binomial regression.

Variables	Parameter Estimation	Std Error	χ2	*P*-value	Main Effect	Total Effect
age	–0.011	0.006	3.875	0.049	0.009	0.017
basal FSH	–0.024	0.010	5.453	0.020	0.014	0.023
log[basal AMH]	0.398	0.041	95.741	<.001	0.878	0.900
basal inhibin B	0.001	0.001	2.299	0.130	0.009	0.015
log[AFC]	0.136	0.053	6.543	0.011	0.033	0.047

Key: NOR, the number of oocytes retrieved; χ2, Chi-square; AMH, anti-Müllerian hormone; FSH, follicle-stimulating hormone; AFC, antral follicle counts.

### Model 2 Using Prospective Data of Basal as Well as Dynamic Levels of Ovarian Reserve Markers to Predict NOR

It is known that the basal ovarian reserve markers reflect the size of the resting ovarian follicle pool (11, 12). Inhibin B is secreted by small FSH-dependent growing follicles 10–12 mm in diameter (13), so reflects activated follicles. Next, we explored whether adding these dynamic predictors would improve the performance of our models in predicting NOR.

After logarithmic transformation of AMH, Δ inhibin B and AFC, multiple negative binomial regression analysis with pruned forward selection and 30% holdback validation was then used to construct Model 2 using both basal and dynamic predictors. The analyzing process and results are shown in **Supplementary Figure 1**. We discovered that when four variables were included, the scaled –*Log* L (*β*) no longer declines, thus the four variables of log[Δinhibin B], log[basal AMH], AFC and age were finally included according to their significances. The main effect of serum Δinhibin B could explain 58.9% of the observed NOR, followed by basal AMH level, which explained 31.6% of the outcome variable, and AFC and age, which explained 4.3% and 0.4% of the outcome variable, respectively. The parameter estimation of Model 2 is also shown in **Table 3**.

**Table 3:** Model-2 using basal predictors to predict the NOR by multiple negative binomial regression.

Variables	Parameter Estimation	Std Error	χ2	*P*-value	Main Effect	Total Effect
log[Δinhibin B]	0.288	0.05	33.456	<.001	0.589	0.615
Log[basal AMH]	0.222	0.046	23.397	<.001	0.316	0.341
log[AFC]	0.144	0.046	9.877	0.002	0.043	0.055
age	–0.007	0.005	1.511	0.219	0.004	0.007

Key: NOR, the number of oocytes retrieved; χ2, Chi-square; Dinhibin B, inhibin B level of day-6 minus day-2; AMH, anti-Müllerian hormone; AFC,antral follicle counts.

### The Performance of Model 1 Using Basal Predictors and Model 2 Using Both Basal and Dynamic Predictors

**Figure 4:**
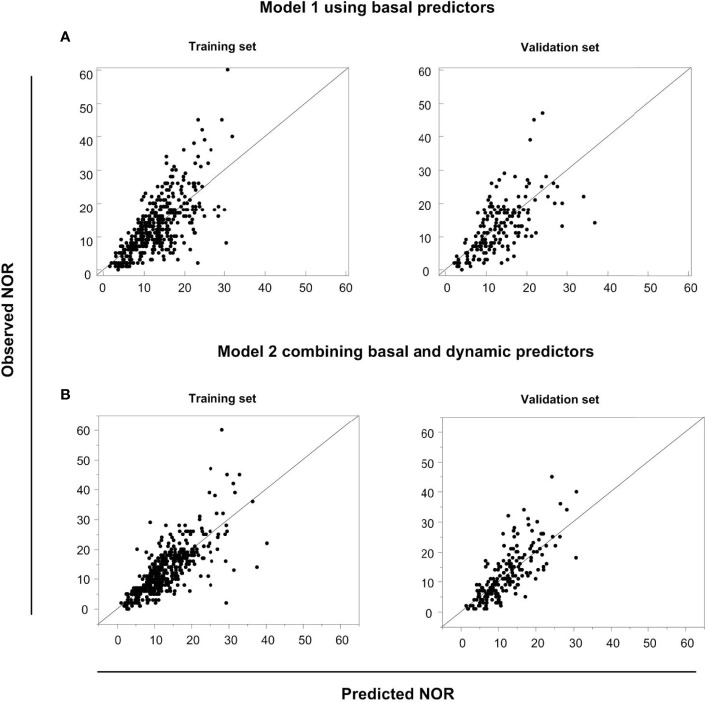
The predictive performances of Models 1 and 2. The relationship between the predicted NOR and the observed NOR in the training and validation sets in Model 1 **(A)** and Model 2 **(B)** are shown graphically.

We compared AICc, BIC, scaled –*Log* L (*β*), and generalized *R* ^2^ values in Models 1 and 2 in the training and validation sets using the same data (**Table 4**). The validation set was used to test the performance of the model established in the training set. The generalized *R* ^2^ value of Model 2 in the validation set was greater than in Model 1, with smaller AICc, BIC and scaled –*Log* L (*β*) values compared with Model 1. We conclude that Model 2 would be better than Model 1 in predicting NOR, if activated ovarian reserve markers are available. To display the predictive effects of Models 1 and 2, the relationships between the predicted and the observed NOR in the training and validation sets are shown in **Figures 4A, B**. The scatter spots were evenly distributed on the diagonal line and its two sides, indicating the accuracy of Models 1 and 2. The actual and predicted NOR are shown in **Supplementary Table 1**. **Figure 4** shows that the performance of Model 2 was better than Model 1, with scatter spots more evenly distributed closer to the diagonal line than Model 1.

**Table 4:** The performance of Model-1 and Model-2 using the same data.

Measure	Model 1		Model 2	
	Training	Validation	Training	Validation
Scaled *–Log* L*(β)*	1253.94	542.97	1214.72	533.08
BIC	2550.10	1122.24	2465.68	1097.36
AICc	2522.14	1100.59	2441.64	1078.65
Generalized R^2^	0.56	0.51	0.64	0.62

Key: –Log L(β), –log[likelihood]; BIC, Schwarz Bayesian information criterion; AICc, corrected Akaike’s information criterion.

**Figure 5:**
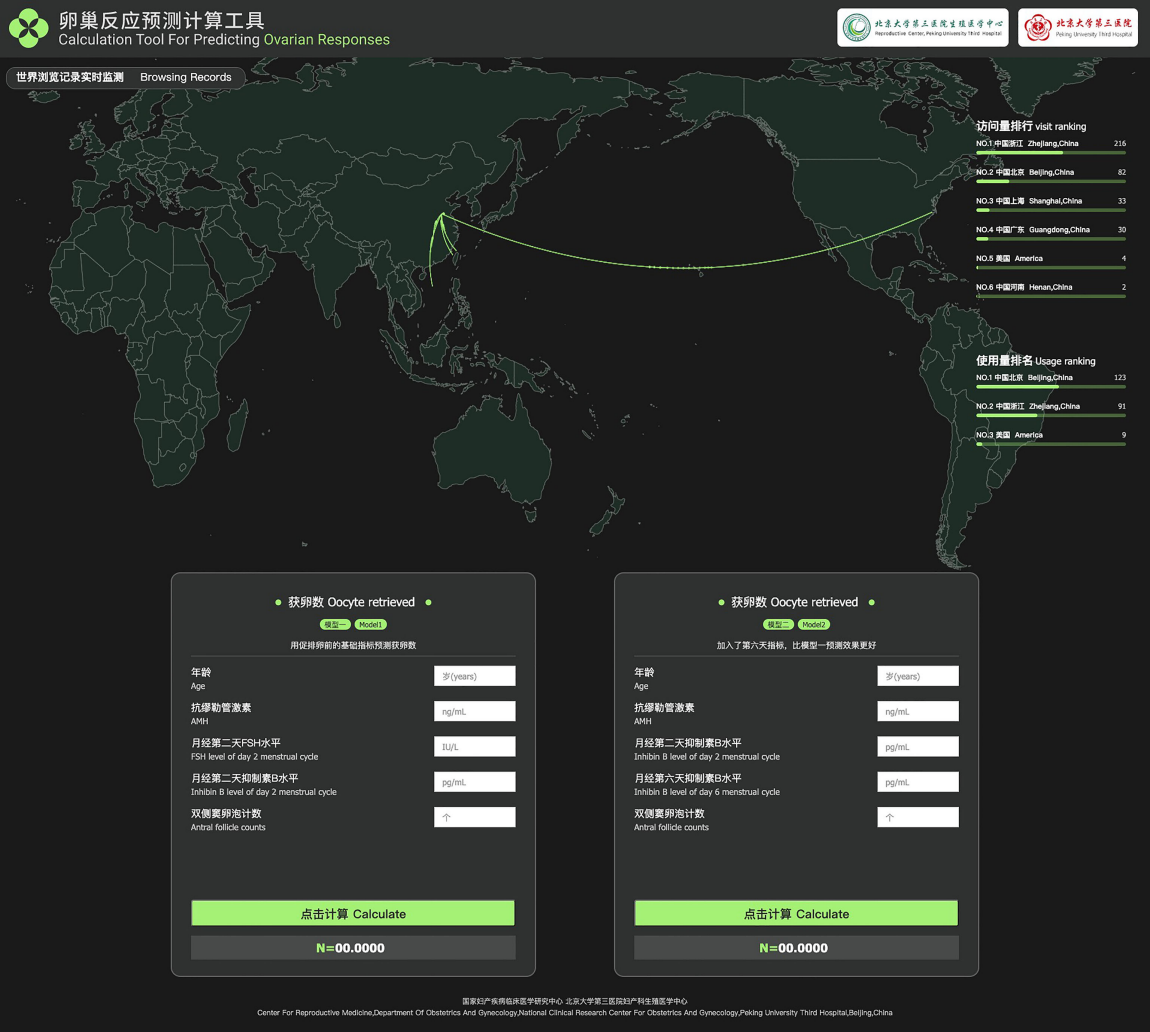
Online calculation tool for predicting NOR.

**Table 5** shows the basic and predicted characteristics of top five cases of having the predicted most and least NOR. An online tool for predicting NOR has been developed for Chinese ART clinics (http://121.43.113.123:8002/). In this tool, users can input the required indicators, click ‘calculate’, and the results of NOR will be returned (**Figure 5**).

**Table 5:** The basic and predicted characteristics of top five cases of having the most and least predicted NOR according to model 2.

Subjects	Age	D2-FSH	AMH	day 2-inhinbinB	Δ inhibin B	AFC	NOR	2PN fertilization rate	embryos available for transfer	predicted NOR by model 1	predicted NOR by model 2
1	37	11.6	0.3	29.27	20.52	3	1	100%	1	4	2
2	45	14.2	0.5	46.31	19.01	5	1	100%	1	4	2
3	39	9.23	0.1	12.35	88.38	3	1	0%	0	2	2
4	31	5.39	0.1	1	3.58	4	2	0%	0	3	1
5	39	13.2	0.1	1	16.08	3	2	0%	0	2	2
6	26	5.05	12.5	108.64	5234.86	24	36	75%	21	30	35
7	26	4.39	15.6	199.06	4540.14	40	22	68%	5	38	37
8	28	4.41	16.5	101.58	3153.92	24	45	49%	18	33	31
9	33	3.58	13.6	87.26	4727.64	18	39	69%	18	29	31
10	33	5.27	24.2	200.52	3308.43	40	14	86%	9	43	36

Key: NOR, the number of oocytes retrieved; D inhibin B, day 6 minus day 2 inhibin B; PN, pronuclear; model 1, predicting NOR using basal predictors; model 2, predicting NOR using both basal and dynamic predictors.

## Discussion

Predicting the NOR prior to COS is the only way to ensure effective and safe ART treatment, and might help contribute to the development of individualized stimulation protocols (1, 4, 14). Here we used basic and dynamic ovarian reserve markers to predict NOR, respectively (Models 1 and 2). The generalized *R* ^2^ value increased significantly from 0.56 and 0.51 in Model 1 to 0.64 and 0.62 in Model 2 in the set and validation sets, respectively. To our knowledge, this is the largest generalized *R* ^2^ value reported among existing models for predicting NOR (15, 16). Model 1 combined with Model 2 could contribute to directing the starting (day 2) dose as well as adjusting the day 6 dose of rFSH during COS, reducing iatrogenic ovarian under- or over-responses, and reducing the cost of ART.

Although the basal ovarian reserve before COS reflects the size of the primordial follicle pool (i.e., the ovarian reserve), there is heterogeneity of ovarian response to exogenous FSH stimulation among women with the same ovarian reserve (17). Therefore, some studies have proposed using dynamic changes in ovarian reserve markers during COS to predict ovarian response (7, 18). Thus, inhibin B has been reported to participate in the selection of follicles in the normal menstrual cycle through endocrine and paracrine effects and promotes FSH-dependent folliculogenesis (19, 20). The secretion of inhibin B reaches its peak in early follicular phase with follicle diameters of 10–12 mm (13). It also exerts a paracrine effect, stimulating the production of androgens and LH from theca cells (13). Moreover, the day 5 (early follicular phase) inhibin B level is a superior marker for both poor ovarian response and LBR compared with basal markers (21). Inhibin B is mainly produced by FSH-sensitive follicles, so administration of exogenous FSH leads to its increase in growing follicles (20). Consistent with this, we discovered that the early Δ inhibin B measure (day 6 minus day 2) was the best marker for predicting NOR, suggesting that this might become a new clinical indicator for monitoring COS. However, because of the nonlinear correlation between independent variables and the outcome variable, complex mathematical transformations are required, so dedicated software will be needed to facilitate the application of this model in ART clinics.

Recombinant follicle-stimulating hormone (rFSH) is most often used during ovarian stimulation. The starting and daily doses of rFSH are important to obtain optimal NOR. Currently, most clinicians still rely on standard doses of rFSH, typically 150–225 IU, adjusted based on their experience and the patients’ previous attempts. However, the heterogeneity in ovarian reserve and responsiveness to rFSH exists, resulting in a large difference in NOR. Therefore, it is very important to predict the NOR prior to or during ovarian stimulation in order to choose appropriate starting or daily dose of rFSH. In the future, we will develop models to predict starting or daily dose of rFSH according to model1 (basic predictors) or model2 (basic and dynamic predictors) respectively, in order to achieve better clinical outcomes and to reduce the costs in IVF cycles.

Logistic regression analysis has been used widely to predict the ovarian response to COS. However, classifying the NOR into two categories is not specific enough for individuals, and the prediction of specific NOR may be of better clinical significance for individualized COS strategies. To our knowledge, publications using mathematical models to predict specific NOR are limited. Lorusso et al. (15) established a Poisson regression to predict NOR in a GnRH agonist COS protocol based on 71 subjects recruited retrospectively. They found that a model using AFC and age provided the best performance. The post-GnRH levels of FSH and E_2_ had no significance in predicting NOR in their protocol (15). Moon et al. (16). recruited 141 cycles of both GnRH antagonist and agonist COS protocols. Poisson regression analysis was also used to predict the NOR. The final predictors of the model were age, basal FSH and AMH levels, and the AFC. In our analysis, a negative binomial distribution was better fitted to our outcome variable of NOR that a Poisson distribution, so this was used in our analysis. As to comparison of generalized *R* ^2^ values in different models, our Model 1 using basal predictors and Model 2 using both basal and dynamic predictors gave values of 0.51 and 0.62 in validation sets, compared with the 0.35 in the model of Lorusso et al. (15) and there was no use of generalized *R* ^2^ in the model of Moon et al. (16). Furthermore, validation of the model was not performed in their papers, possibly because of insufficient sample size. Moreover, the COS protocols also differed from our study as GnRH-agonist cycles were included in both previous studies. We included only GnRH-antagonist cycles because the hormone levels in this population reflects the general population, so our Model 1 could be applied to any assessment prior to ovarian stimulation for individualized COS in the future.

Whether AMH or AFC is better for predicting ovarian response has been a controversial issue, with different conclusions drawn from different studies in different populations (22–24). Our results suggest that the significance of AMH is superior to AFC, with main effects of 87.8% and 3.3% in Model 1 and 31.6% and 4.3% in Model 2, respectively. First, one possible reason why AMH is superior to AFC might be that the accuracy and repeatability of commercial AMH tests are satisfactory (25), but for AFC estimates there are many interfering factors. Even in single-center studies such as ours, although the definition of AFC and the instruments of ultrasonography are standardized, the AFC is still heavily influenced by the heterogeneity of the skills of individual clinicians who perform the measurements. Second, considering the high correlation between AFC and AMH, a large contribution of AFC in predicting NOR could be replaced by the use of AMH levels.

There were limitations to our study. First, we did not conduct data selection and the inclusion of patients with histories of clinical factors such as polycystic ovarian syndrome or ovarian surgery could affect the performance of the models in certain cases. However, our models might have a wider application in the general population. Our mathematical model will be developed into software or a smartphone app for doctor-side monitoring of follicular growth. In the future, through the collection of more retrospective data, we will further optimize the model by taking into consideration different causes of infertility. Second, this study used the Ansh laboratory ELISA kit for detecting serum levels of inhibin B. If our Model 2 is to be clinically applied in the future, a chemiluminescence kit would be beneficial, but heterogeneity of results from different assay platforms still needs attention.

In conclusion, to achieve good efficacy and safety in a relatively homogenous population, individualized COS is the optimal choice (26, 27). We have established Model 1 based on basic ovarian reserve markers and Model 2 based on both basic and dynamic predictors. We hope that the combination of two models could contribute to better directing of both the rFSH starting dose and adjusting this during COS, which could help in reducing iatrogenic ovarian under- or over responses, and in reducing the cost of ART. We are now developing these mathematical models into software for promoting a tailored approach to patient management before and during COS, and to identify more homogeneous populations, thereby providing better tools with which to maximize ART success rates.

## Data Availability Statement

The original contributions presented in the study are included in the article/**Supplementary Material**. Further inquiries can be directed to the corresponding authors.

## Ethics Statement

The studies involving human participants were reviewed and approved by The institutional review board of Peking University Third Hospital. The patients/participants provided their written informed consent to participate in this study.

## Author Contributions

YH: data analysis and manuscript writing. HX: data collection and manuscript writing. GF: statistical analysis, and manuscript writing. KA: statistical analysis, and manuscript writing. LC: data collection. HW: data collection and manuscript writing. RL: resources, study design, supervision and final manuscript approval. All authors contributed to the article and approved the submitted version.

## Funding

This study was supported by the National Natural Science Foundation of China for Distinguished Young Scholars (Grant No. 81925013); the Innovation & Transfer Fund of Peking University Third Hospital (Grant No. BYSYZHZB2020102); Major National R&D Projects of China (Grant No. 2017ZX09304012-012); the National Natural Science Foundation of China (Grant No. 81771650); and the Capital Health Research and Development of Special Project (Grant No. 2018-1-4091).

## Conflict of Interest

Author KA is employed by The Predict Health.

The remaining authors declare that the research was conducted in the absence of any commercial or financial relationships that could be construed as a potential conflict of interest.

## Publisher’s Note

All claims expressed in this article are solely those of the authors and do not necessarily represent those of their affiliated organizations, or those of the publisher, the editors and the reviewers. Any product that may be evaluated in this article, or claim that may be made by its manufacturer, is not guaranteed or endorsed by the publisher.
