# Supplementary material for: An Online Tool Using Basal or Activated Ovarian Reserve Markers to Predict the Number of Oocytes Retrieved Following Controlled Ovarian Stimulation: A Prospective Observational Cohort Study
Source: Front Endocrinol (Lausanne). 2022 May 27;13:881983. doi: 10.3389/fendo.2022.881983 (PMC9186016; doi:10.3389/fendo.2022.881983)
Supplement: Supplementary file 1 [file DataSheet_1.docx]

**Supplementary materials**

Supplementary Figure 1


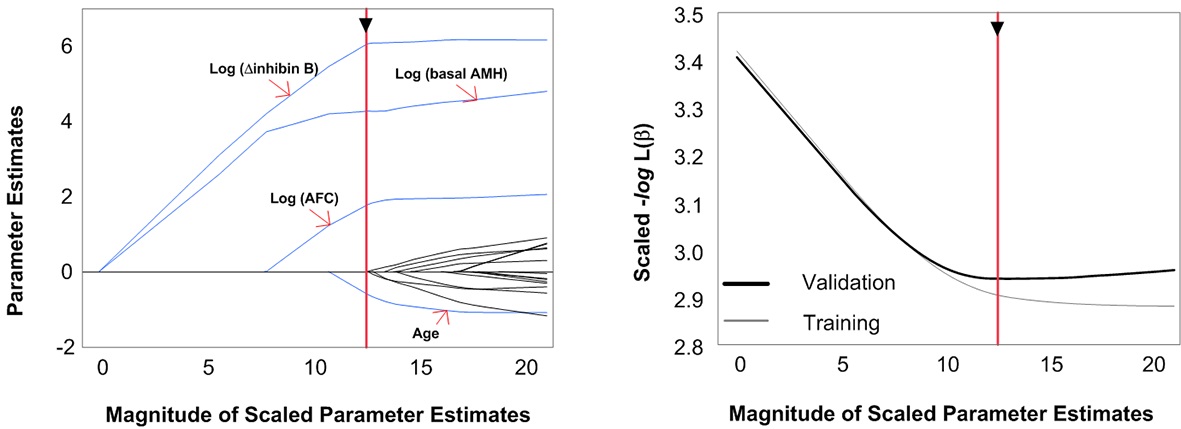


Supplementary Table 1. The actual and predicted NOR.

| Actual NORs | predicted NORs using model 1 | predicted NORs using model 2 |
| --- | --- | --- |
| 9 | 10 | 8 |
| 3 | 8 | 6 |
| 9 | 10 | 9 |
| 25 | 23 | 19 |
| 7 | 11 | 10 |
| 1 | 2 | 2 |
| 7 | 5 | 5 |
| 13 | 14 | 16 |
| 5 | 4 | 3 |
| 10 | 11 | 10 |
| 6 | 9 | 8 |
| 5 | 5 | 5 |
| 7 | 14 | 11 |
| 10 | 22 | 13 |
| 20 | 14 | 17 |
| 14 | 11 | 10 |
| 19 | 13 | 13 |
| 12 | 13 | 11 |
| 15 | 19 | 17 |
| 18 | 22 | 17 |
| 11 | 18 | 15 |
| 13 | 21 | 17 |
| 4 | 6 | 6 |
| 12 | 10 | 9 |
| 0 | 4 | 3 |
| 5 | 12 | 10 |
| 6 | 9 | 8 |
| 20 | 18 | 17 |
| 7 | 8 | 6 |
| 11 | 12 | 12 |
| 26 | 30 | 28 |
| 9 | 12 | 12 |
| 1 | 4 | 2 |
| 12 | 9 | 9 |
| 6 | 14 | 12 |
| 2 | 6 | 4 |
| 6 | 9 | 9 |
| 9 | 5 | 4 |
| 4 | 8 | 6 |
| 18 | 14 | 13 |
| 13 | 11 | 11 |
| 4 | 3 | 3 |
| 9 | 15 | 14 |
| 36 | 30 | 35 |
| 26 | 18 | 18 |
| 21 | 24 | 24 |
| 11 | 12 | 10 |
| 20 | 13 | 15 |
| 18 | 25 | 19 |
| 8 | 6 | 6 |
| 7 | 19 | 12 |
| 10 | 9 | 8 |
| 28 | 19 | 18 |
| 7 | 6 | 7 |
| 17 | 20 | 18 |
| 13 | 14 | 13 |
| 11 | 13 | 10 |
| 5 | 13 | 10 |
| 18 | 18 | 18 |
| 9 | 16 | 15 |
| 10 | 12 | 9 |
| 36 | 23 | 26 |
| 18 | 16 | 15 |
| 22 | 13 | 12 |
| 6 | 19 | 14 |
| 21 | 25 | 23 |
| 7 | 12 | 9 |
| 12 | 14 | 11 |
| 11 | 21 | 16 |
| 22 | 22 | 24 |
| 2 | 8 | 6 |
| 2 | 4 | 3 |
| 7 | 11 | 9 |
| 15 | 10 | 9 |
| 4 | 11 | 8 |
| 5 | 11 | 9 |
| 3 | 8 | 7 |
| 12 | 26 | 17 |
| 10 | 11 | 10 |
| 39 | 24 | 24 |
| 5 | 19 | 17 |
| 19 | 15 | 8 |
| 28 | 23 | 14 |
| 4 | 3 | 4 |
| 12 | 19 | 17 |
| 13 | 14 | 13 |
| 7 | 9 | 7 |
| 9 | 9 | 10 |
| 16 | 11 | 10 |
| 15 | 17 | 13 |
| 45 | 27 | 28 |
| 16 | 28 | 29 |
| 30 | 20 | 20 |
| 15 | 12 | 10 |
| 13 | 12 | 10 |
| 7 | 12 | 8 |
| 14 | 24 | 21 |
| 7 | 7 | 6 |
| 8 | 11 | 8 |
| 3 | 17 | 9 |
| 15 | 15 | 13 |
| 17 | 21 | 15 |
| 13 | 11 | 11 |
| 12 | 12 | 11 |
| 5 | 11 | 10 |
| 13 | 17 | 18 |
| 25 | 17 | 18 |
| 7 | 11 | 9 |
| 14 | 16 | 15 |
| 22 | 29 | 25 |
| 17 | 17 | 16 |
| 18 | 19 | 15 |
| 7 | 8 | 8 |
| 11 | 12 | 11 |
| 16 | 21 | 19 |
| 23 | 20 | 19 |
| 9 | 14 | 12 |
| 6 | 6 | 5 |
| 7 | 12 | 11 |
| 8 | 11 | 9 |
| 2 | 7 | 5 |
| 21 | 15 | 14 |
| 11 | 20 | 20 |
| 14 | 21 | 20 |
| 32 | 26 | 26 |
| 19 | 26 | 21 |
| 14 | 12 | 11 |
| 6 | 18 | 10 |
| 20 | 24 | 21 |
| 12 | 17 | 15 |
| 4 | 19 | 10 |
| 15 | 14 | 15 |
| 27 | 22 | 18 |
| 17 | 13 | 12 |
| 11 | 16 | 11 |
| 5 | 8 | 7 |
| 17 | 15 | 18 |
| 6 | 7 | 9 |
| 9 | 10 | 10 |
| 13 | 15 | 13 |
| 27 | 15 | 14 |
| 7 | 8 | 8 |
| 17 | 9 | 9 |
| 19 | 17 | 17 |
| 27 | 23 | 22 |
| 2 | 3 | 4 |
| 17 | 14 | 12 |
| 16 | 17 | 7 |
| 3 | 10 | 9 |
| 9 | 10 | 10 |
| 3 | 8 | 5 |
| 6 | 8 | 7 |
| 3 | 6 | 4 |
| 14 | 10 | 10 |
| 26 | 25 | 21 |
| 4 | 8 | 7 |
| 19 | 21 | 16 |
| 8 | 8 | 7 |
| 16 | 18 | 17 |
| 26 | 18 | 17 |
| 47 | 27 | 24 |
| 20 | 15 | 15 |
| 18 | 20 | 19 |
| 17 | 19 | 17 |
| 11 | 26 | 15 |
| 7 | 12 | 8 |
| 17 | 21 | 22 |
| 11 | 7 | 7 |
| 14 | 19 | 15 |
| 13 | 12 | 9 |
| 13 | 12 | 9 |
| 13 | 12 | 10 |
| 18 | 35 | 30 |
| 2 | 3 | 1 |
| 1 | 4 | 2 |
| 10 | 13 | 13 |
| 14 | 24 | 17 |
| 12 | 16 | 15 |
| 7 | 10 | 9 |
| 17 | 17 | 15 |
| 23 | 13 | 13 |
| 18 | 16 | 16 |
| 26 | 21 | 22 |
| 17 | 15 | 16 |
| 6 | 9 | 8 |
| 11 | 11 | 11 |
| 17 | 14 | 11 |
| 45 | 33 | 31 |
| 0 | 5 | 3 |
| 26 | 18 | 15 |
| 18 | 14 | 14 |
| 12 | 16 | 15 |
| 14 | 16 | 16 |
| 9 | 14 | 15 |
| 19 | 13 | 14 |
| 11 | 16 | 12 |
| 17 | 19 | 17 |
| 22 | 18 | 16 |
| 15 | 12 | 11 |
| 16 | 11 | 10 |
| 15 | 11 | 10 |
| 18 | 24 | 18 |
| 1 | 7 | 7 |
| 14 | 13 | 12 |
| 8 | 12 | 9 |
| 10 | 16 | 13 |
| 15 | 18 | 17 |
| 12 | 18 | 16 |
| 38 | 26 | 26 |
| 10 | 13 | 13 |
| 22 | 16 | 15 |
| 6 | 20 | 19 |
| 7 | 13 | 11 |
| 17 | 15 | 17 |
| 5 | 5 | 3 |
| 7 | 8 | 7 |
| 12 | 15 | 12 |
| 9 | 8 | 7 |
| 24 | 22 | 14 |
| 8 | 20 | 11 |
| 11 | 10 | 8 |
| 10 | 16 | 13 |
| 13 | 20 | 18 |
| 17 | 15 | 13 |
| 16 | 10 | 8 |
| 7 | 7 | 6 |
| 7 | 13 | 9 |
| 9 | 10 | 10 |
| 16 | 13 | 12 |
| 19 | 21 | 21 |
| 8 | 7 | 9 |
| 10 | 13 | 12 |
| 25 | 27 | 28 |
| 10 | 36 | 15 |
| 10 | 8 | 8 |
| 18 | 16 | 15 |
| 3 | 13 | 10 |
| 15 | 14 | 12 |
| 8 | 20 | 14 |
| 14 | 13 | 12 |
| 20 | 32 | 26 |
| 13 | 11 | 12 |
| 13 | 15 | 13 |
| 60 | 36 | 28 |
| 8 | 15 | 15 |
| 3 | 10 | 10 |
| 13 | 20 | 18 |
| 25 | 22 | 17 |
| 13 | 11 | 11 |
| 17 | 15 | 13 |
| 11 | 12 | 9 |
| 8 | 12 | 11 |
| 18 | 15 | 15 |
| 1 | 4 | 3 |
| 5 | 6 | 4 |
| 14 | 18 | 14 |
| 15 | 11 | 7 |
| 17 | 11 | 11 |
| 2 | 7 | 6 |
| 10 | 12 | 11 |
| 19 | 18 | 20 |
| 28 | 29 | 29 |
| 11 | 10 | 8 |
| 17 | 14 | 13 |
| 6 | 15 | 11 |
| 29 | 23 | 18 |
| 10 | 19 | 16 |
| 5 | 6 | 5 |
| 10 | 20 | 10 |
| 8 | 8 | 8 |
| 14 | 16 | 14 |
| 6 | 6 | 5 |
| 5 | 6 | 5 |
| 16 | 11 | 9 |
| 7 | 10 | 9 |
| 11 | 13 | 13 |
| 15 | 10 | 10 |
| 18 | 13 | 13 |
| 19 | 16 | 17 |
| 14 | 11 | 10 |
| 3 | 6 | 5 |
| 6 | 17 | 14 |
| 11 | 15 | 15 |
| 8 | 9 | 8 |
| 11 | 10 | 9 |
| 11 | 11 | 9 |
| 7 | 9 | 6 |
| 24 | 17 | 12 |
| 3 | 10 | 8 |
| 25 | 26 | 24 |
| 17 | 21 | 18 |
| 21 | 17 | 15 |
| 13 | 16 | 15 |
| 8 | 16 | 12 |
| 14 | 14 | 13 |
| 12 | 15 | 12 |
| 5 | 8 | 7 |
| 4 | 7 | 6 |
| 20 | 13 | 15 |
| 7 | 8 | 8 |
| 11 | 11 | 10 |
| 15 | 20 | 21 |
| 12 | 11 | 10 |
| 8 | 15 | 8 |
| 15 | 16 | 13 |
| 4 | 6 | 5 |
| 12 | 14 | 10 |
| 10 | 20 | 10 |
| 7 | 16 | 9 |
| 7 | 13 | 13 |
| 6 | 26 | 16 |
| 21 | 17 | 21 |
| 5 | 16 | 10 |
| 19 | 33 | 24 |
| 16 | 13 | 14 |
| 10 | 18 | 15 |
| 12 | 17 | 10 |
| 9 | 16 | 14 |
| 11 | 11 | 10 |
| 14 | 16 | 12 |
| 17 | 23 | 12 |
| 16 | 15 | 10 |
| 11 | 14 | 13 |
| 10 | 13 | 12 |
| 2 | 7 | 4 |
| 9 | 16 | 14 |
| 9 | 11 | 9 |
| 14 | 10 | 9 |
| 25 | 25 | 26 |
| 2 | 11 | 8 |
| 2 | 6 | 5 |
| 11 | 12 | 11 |
| 20 | 14 | 16 |
| 8 | 9 | 9 |
| 15 | 13 | 13 |
| 9 | 11 | 8 |
| 45 | 25 | 24 |
| 20 | 19 | 18 |
| 3 | 6 | 3 |
| 7 | 9 | 8 |
| 19 | 15 | 15 |
| 30 | 20 | 22 |
| 16 | 20 | 13 |
| 14 | 15 | 13 |
| 25 | 32 | 22 |
| 9 | 10 | 9 |
| 8 | 34 | 24 |
| 5 | 11 | 10 |
| 6 | 10 | 8 |
| 13 | 13 | 9 |
| 4 | 7 | 5 |
| 5 | 5 | 4 |
| 11 | 16 | 14 |
| 13 | 18 | 17 |
| 9 | 19 | 15 |
| 21 | 12 | 10 |
| 2 | 5 | 4 |
| 6 | 6 | 5 |
| 13 | 10 | 10 |
| 14 | 12 | 11 |
| 5 | 8 | 5 |
| 16 | 23 | 25 |
| 14 | 17 | 16 |
| 6 | 15 | 11 |
| 16 | 32 | 22 |
| 21 | 16 | 13 |
| 11 | 17 | 12 |
| 5 | 11 | 7 |
| 19 | 17 | 17 |
| 8 | 11 | 9 |
| 2 | 4 | 3 |
| 16 | 23 | 18 |
| 18 | 16 | 16 |
| 10 | 17 | 10 |
| 18 | 18 | 19 |
| 24 | 26 | 26 |
| 1 | 7 | 6 |
| 28 | 19 | 16 |
| 10 | 9 | 9 |
| 16 | 19 | 15 |
| 26 | 20 | 21 |
| 5 | 11 | 9 |
| 19 | 17 | 15 |
| 2 | 7 | 5 |
| 2 | 2 | 2 |
| 3 | 8 | 6 |
| 15 | 18 | 15 |
| 9 | 11 | 10 |
| 1 | 5 | 3 |
| 31 | 28 | 22 |
| 18 | 19 | 16 |
| 4 | 11 | 8 |
| 7 | 6 | 6 |
| 15 | 25 | 21 |
| 12 | 16 | 13 |
| 1 | 5 | 3 |
| 5 | 12 | 7 |
| 9 | 12 | 9 |
| 19 | 18 | 16 |
| 11 | 13 | 10 |
| 14 | 14 | 15 |
| 15 | 17 | 13 |
| 6 | 10 | 7 |
| 18 | 29 | 19 |
| 5 | 6 | 5 |
| 2 | 4 | 3 |
| 9 | 15 | 14 |
| 19 | 22 | 18 |
| 9 | 20 | 13 |
| 24 | 19 | 21 |
| 13 | 12 | 10 |
| 16 | 24 | 20 |
| 2 | 4 | 3 |
| 8 | 23 | 17 |
| 16 | 13 | 11 |
| 42 | 28 | 30 |
| 2 | 16 | 10 |
| 29 | 17 | 9 |
| 1 | 7 | 6 |
| 6 | 15 | 14 |
| 8 | 11 | 8 |
| 21 | 25 | 23 |
| 2 | 4 | 3 |
| 7 | 5 | 5 |
| 3 | 8 | 6 |
| 2 | 3 | 3 |
| 6 | 6 | 5 |
| 4 | 9 | 5 |
| 13 | 15 | 13 |
| 10 | 13 | 11 |
| 8 | 16 | 18 |
| 9 | 11 | 11 |
| 8 | 17 | 10 |
| 6 | 10 | 10 |
| 8 | 7 | 6 |
| 3 | 4 | 3 |
| 6 | 8 | 6 |
| 3 | 11 | 7 |
| 18 | 16 | 12 |
| 22 | 20 | 18 |
| 2 | 7 | 5 |
| 1 | 4 | 1 |
| 4 | 17 | 10 |
| 15 | 14 | 10 |
| 2 | 10 | 7 |
| 5 | 19 | 15 |
| 12 | 16 | 16 |
| 11 | 15 | 9 |
| 16 | 15 | 15 |
| 17 | 15 | 15 |
| 25 | 15 | 14 |
| 7 | 7 | 5 |
| 26 | 25 | 24 |
| 11 | 12 | 8 |
| 20 | 27 | 19 |
| 19 | 16 | 15 |
| 2 | 27 | 29 |
| 19 | 25 | 18 |
| 11 | 12 | 11 |
| 20 | 23 | 20 |
| 1 | 5 | 4 |
| 18 | 26 | 20 |
| 7 | 18 | 14 |
| 18 | 17 | 16 |
| 0 | 4 | 3 |
| 9 | 11 | 9 |
| 17 | 21 | 18 |
| 9 | 12 | 9 |
| 28 | 20 | 13 |
| 17 | 22 | 13 |
| 11 | 13 | 11 |
| 2 | 9 | 6 |
| 11 | 12 | 11 |
| 11 | 18 | 9 |
| 10 | 9 | 7 |
| 14 | 16 | 14 |
| 9 | 13 | 10 |
| 9 | 16 | 12 |
| 7 | 16 | 9 |
| 23 | 16 | 15 |
| 7 | 10 | 8 |
| 22 | 23 | 23 |
| 13 | 33 | 30 |
| 6 | 7 | 7 |
| 18 | 11 | 10 |
| 2 | 7 | 6 |
| 17 | 26 | 23 |
| 14 | 12 | 11 |
| 11 | 25 | 21 |
| 8 | 8 | 8 |
| 5 | 6 | 4 |
| 10 | 21 | 16 |
| 5 | 6 | 6 |
| 2 | 3 | 2 |
| 7 | 9 | 7 |
| 6 | 13 | 9 |
| 10 | 14 | 12 |
| 26 | 23 | 19 |
| 16 | 12 | 12 |
| 2 | 4 | 3 |
| 3 | 5 | 4 |
| 1 | 8 | 7 |
| 13 | 18 | 13 |
| 9 | 16 | 11 |
| 28 | 21 | 15 |
| 18 | 20 | 14 |
| 18 | 32 | 16 |
| 31 | 20 | 18 |
| 3 | 14 | 10 |
| 9 | 17 | 12 |
| 7 | 9 | 7 |
| 15 | 11 | 14 |
| 10 | 22 | 15 |
| 10 | 17 | 14 |
| 21 | 12 | 12 |
| 3 | 11 | 9 |
| 12 | 12 | 10 |
| 20 | 22 | 19 |
| 17 | 19 | 16 |
| 13 | 13 | 10 |
| 10 | 14 | 12 |
| 26 | 26 | 21 |
| 1 | 6 | 4 |
| 7 | 7 | 6 |
| 16 | 16 | 13 |
| 26 | 24 | 21 |
| 7 | 12 | 10 |
| 25 | 19 | 23 |
| 16 | 16 | 17 |
| 3 | 12 | 9 |
| 8 | 15 | 11 |
| 14 | 43 | 36 |
| 26 | 13 | 11 |
| 6 | 10 | 7 |
| 7 | 10 | 11 |
| 22 | 38 | 37 |
| 5 | 6 | 5 |
| 8 | 10 | 9 |
| 34 | 27 | 27 |
| 18 | 23 | 21 |
| 18 | 23 | 19 |
| 15 | 14 | 13 |
| 32 | 11 | 11 |
| 25 | 16 | 15 |
| 18 | 16 | 14 |
| 16 | 12 | 12 |
| 22 | 11 | 14 |
| 15 | 24 | 19 |
| 2 | 4 | 3 |
| 32 | 17 | 12 |
| 16 | 14 | 13 |
| 20 | 31 | 20 |
| 5 | 14 | 11 |
| 20 | 18 | 5 |
| 23 | 22 | 18 |
| 22 | 20 | 18 |
| 14 | 19 | 16 |
| 8 | 14 | 11 |
| 18 | 22 | 18 |
| 11 | 26 | 24 |
| 21 | 21 | 18 |
| 21 | 18 | 21 |
| 12 | 17 | 15 |
| 25 | 28 | 26 |
| 17 | 15 | 17 |
| 8 | 13 | 11 |
| 1 | 4 | 2 |
| 8 | 9 | 8 |
| 12 | 15 | 14 |
| 15 | 19 | 19 |
| 6 | 8 | 5 |
| 5 | 14 | 12 |
| 13 | 22 | 17 |
| 8 | 8 | 6 |
| 6 | 10 | 9 |
| 11 | 18 | 12 |
| 4 | 7 | 6 |
| 18 | 20 | 18 |
| 22 | 14 | 12 |
| 10 | 24 | 13 |
| 7 | 5 | 5 |
| 9 | 11 | 10 |
| 1 | 5 | 2 |
| 1 | 4 | 3 |
| 3 | 17 | 11 |
| 17 | 19 | 18 |
| 19 | 13 | 13 |
| 12 | 23 | 18 |
| 1 | 6 | 4 |
| 14 | 13 | 12 |
| 16 | 15 | 16 |
| 17 | 14 | 6 |
| 39 | 29 | 31 |
| 26 | 18 | 19 |
| 19 | 27 | 15 |
| 5 | 12 | 11 |
| 15 | 16 | 16 |
| 8 | 19 | 12 |
| 4 | 7 | 6 |
| 12 | 10 | 9 |
| 17 | 15 | 14 |
| 6 | 18 | 15 |
| 5 | 8 | 6 |
| 40 | 36 | 29 |
| 34 | 18 | 17 |
| 19 | 18 | 21 |
| 5 | 5 | 5 |
| 32 | 30 | 28 |
| 2 | 11 | 11 |
| 14 | 11 | 10 |
| 21 | 11 | 14 |
| 15 | 12 | 12 |
| 9 | 6 | 5 |
| 15 | 23 | 13 |
| 8 | 13 | 11 |
| 18 | 22 | 13 |
| 7 | 8 | 5 |
| 10 | 15 | 13 |
| 3 | 21 | 11 |
| 7 | 17 | 9 |
| 9 | 14 | 11 |
